# Conflict exposure throughout the life-course and mental health among older adults in Lebanon

**DOI:** 10.1093/geroni/igaf122.508

**Published:** 2025-12-31

**Authors:** Carlos Mendes de Leon, Abla Sibai, Martine Elbejjani, Alexandra Abi Nassif, Mayssan Kabalan, Che Henry Ngwa, Stephen McCall, Monique Chaaya

**Affiliations:** Georgetown University, Washington, District of Columbia, United States; American University of Beirut, Beirut, Beyrouth, Lebanon; American University of Beirut, Beirut, Beyrouth, Lebanon; American University of Beirut, Beirut, Beyrouth, Lebanon; American University of Beirut, Beirut, Beyrouth, Lebanon; American University of Beirut, Beirut, Beyrouth, Lebanon; American University of Beirut, Beirut, Beyrouth, Lebanon; American University of Beirut, Beirut, Beyrouth, Lebanon

## Abstract

Lebanon has experienced a prolonged period of political instability and conflict, starting with the onset of the civil war in 1975. Most older adults in Lebanon have been exposed to conflict, violence and losses during most of their life. We examine the mental health associations of these conflict-related exposures, using baseline data from the Lebanon Study of Aging and HeAlth (LSAHA), a population-based survey of 3,027 adults aged > 60 years (69% participation rate). The sample was obtained using a multi-stage random sampling design in two pre-selected areas: the city of Beirut and the Zahle district (southern Beqaa Valley governate). Conflict exposures were summarized in experiences of personal bodily harm (13.3%), bodily harm to close family members (27.4%), loss of own possessions (e.g., houses, jobs, 35.5%), and loss of possessions among close family members (15.7%). After accounting for the complex sampling design, weighted linear regression models controlling for age, sex, education, and sampling area showed that each unit increase in all four conflict exposure variables was significantly (p’s < .01) associated with higher levels of depressive symptoms (CES-8) and anxiety symptoms (GAD-7). The strongest association for depression was found for experiences of personal bodily harm (ß=1.36, p<.005), while the strongest association for anxiety was for loss of possessions among family members (ß=1.42, p<.001). The results suggest that the high prevalence of conflict-related exposures, whether they resulted in harm or losses to self or to family members, has a substantial adverse effect on the well-being of older adults in Lebanon.

